# Combined microRNA and mRNA detection in mammalian retinas by *in situ* hybridization chain reaction

**DOI:** 10.1038/s41598-019-57194-0

**Published:** 2020-01-15

**Authors:** Pei Zhuang, Huanqing Zhang, Ryan M. Welchko, Robert C. Thompson, Shunbin Xu, David L. Turner

**Affiliations:** 1 Michigan Neuroscience Institute, University of Michigan, Ann Arbor, MI 48109 USA; 2 Department of Psychiatry, University of Michigan, Ann Arbor, MI 48109 USA; 3 Department of Ophthalmology, Visual and Anatomical Sciences, School of Medicine, Wayne State University, Detroit, MI 48201 USA; 4 Department of Biological Chemistry, University of Michigan, Ann Arbor, MI 48109 USA; 5 Present Address: Department of Ophthalmology, Stanford University School of Medicine, Palo Alto, CA 94304 USA; 6 Present Address: Neuroscience Program, College of Science and Engineering, Central Michigan University, Mt. Pleasant, MI 48859 USA

**Keywords:** miRNAs, Cell type diversity

## Abstract

Improved *in situ* hybridization methods for mRNA detection in tissues have been developed based on the hybridization chain reaction (HCR). We show that *in situ* HCR methods can be used for the detection of microRNAs in tissue sections from mouse retinas. *In situ* HCR can be used for the detection of two microRNAs simultaneously or for the combined detection of microRNA and mRNA. In addition, miRNA *in situ* HCR can be combined with immunodetection of proteins. We use these methods to characterize cells expressing specific microRNAs in the mouse retina. We find that miR-181a is expressed in amacrine cells during development and in adult retinas, and it is present in both GABAergic and glycinergic amacrine cells. The detection of microRNAs with *in situ* HCR should facilitate studies of microRNA function and gene regulation in the retina and other tissues.

## Introduction

New methods for analyzing and visualizing gene expression with single cell resolution are facilitating analysis of gene function and development^1–3^. microRNAs (miRNAs) are small RNAs (~21–22 nucleotides: nt) that regulate genes through modulating mRNA stability or translation^4^. In the retina, miRNAs have essential roles both during development and in the adult retina^5^. However, analyzing miRNA expression at the single cell level remains challenging. We^6^ and others^7–11^ have developed *in situ* hybridization methods for detection of endogenous miRNAs in cells or tissue (reviewed in^12^). The short length of mature miRNAs constrains probe size and detection sensitivity. The existence of miRNA families with closely related sequences also complicates the specificity of miRNA detection, but the use of locked nucleic acid (LNA) probes^7^ or high stringency wash conditions^6^ can discriminate between related miRNAs. Most miRNA *in situ* hybridization studies have relied on colorimetric enzyme assays for amplification and detection, but detection methods based on fluorescent enzymatic assays^10,11,13–16^ or radioactive labels^17^ have been employed. Fluorescent detection allows combinatorial detection with fluorescent probes for mRNAs^16^ or antibodies^15^.

The hybridization chain reaction (HCR) is a nucleic acid-based amplification system^18^ that has been applied to the detection of specific RNA or DNA sequences in a wide variety of assays. In HCR, a pair of labeled metastable DNA hairpin molecules (amplifiers) will polymerize only in the presence of specific DNA sequences (initiators). A DNA probe fused to one or more initiator sequences is hybridized to a target molecule. Subsequent incubation with the DNA hairpin amplifiers leads to polymerization and the accumulation of label (usually a fluorescent dye) at the probe binding site. HCR methods for mRNA *in situ* hybridization (*in situ* HCR) have been developed for cells, tissues, or whole embryos^19–23^ and offer advantages over other ISH methods. The amplification/detection reactions are rapid and can be performed under mild conditions, while probes with different initiators can activate orthogonal hairpin amplifiers with distinct fluorescent labels, allowing simplified and simultaneous multiplex detection of different mRNAs. HCR has been used for the detection of miRNAs on northern blots^24^, in solution^25,26^, and recently in tissue culture cells^27^, but HCR has not been applied to the *in situ* detection of miRNA expressing cells in tissue.

Here we demonstrate detection of endogenous miRNAs in tissue sections of mouse retinas by *in situ* HCR. We show that miRNA *in situ* HCR can be used to detect two different miRNAs simultaneously, and that miRNA *in situ* HCR can be combined with *in situ* HCR detection of mRNAs or with immunohistochemical detection of a protein. Combinatorial detection of miRNAs and mRNAs or proteins should facilitate studies of miRNA gene regulation and the identification of miRNA expressing cells in tissues. We use these methods to detect various retinal miRNAs both during development and in the adult. We use markers to characterize retinal cells expressing miR-182, miR-124, and miR-181a. We show that miR-181a is expressed in at least two subpopulations of retinal amacrine cells.

## Results

### *In situ* HCR detection of miRNAs

**Figure 1:**
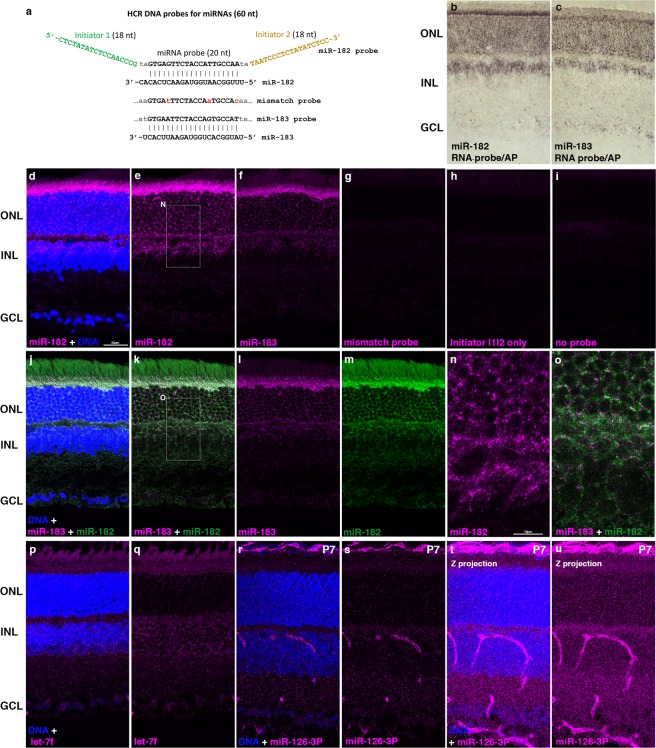
miRNA detection by *in situ* HCR in tissue sections from mouse retinas. (**a**) Schematic of HCR probes for miRNA detection (also see Supplementary Fig. S1); initiator sequences are not shown for the mismatch or miR-183 probes. (**b**,**c**) Non-HCR miRNA *in situ* hybridization for miR-182 or miR-183, using 20 nt fluorescein-labeled RNA probes detected with alkaline phosphatase (AP) conjugated anti-fluorescein antibody (purple). (**d**–**f**, N) miRNA *in situ* HCR for miR-182 or miR-183, (**g**) Probe with 3 mismatches to both miR-182 and miR-183, (**h**) Probe containing only two initiator sequences, or (**i**) No probe added. (**j**–**m**,**o**) Combined detection of miR-182 and miR-183 by *in situ* HCR with two different HCR amplifiers. (**n**,**o**) enlargements of regions indicated by dashed rectangles in (**e**,**k**). (**p**,**q**) miRNA *in situ* HCR for Let-7f or (**r**–**u**) miR-126-3p. **(t**,**u**) Maximum intensity Z projection of miR-126-3p signal across Z-stack (same retina section), showing labeling of retinal blood vessels and choroid. (**b–q**) adult, (**r**–**u**) P7 retinas. Magenta: Cy3, green: Cy 5, blue: DNA in nuclei. Scale bars: 25 µm (**d**–**m**); 10 µm (**n**,**o**).

For HCR detection, a probe sequence is linked to one or more DNA initiator sequences that allow interaction with the labeled metastable hairpin amplifiers used for detection. The mRNA *in situ* HCR methods developed by Pierce and colleagues use 36 nt initiator sequences^19,21^. As the length of the miRNA targets limits miRNA probes to about 20 nt, we wanted to attach HCR initiator sequences of similar length, to minimize nonspecific binding of the initiator sequences during probe hybridization and washing. We based our HCR miRNA probes on a system for multiplex mRNA *in situ* HCR recently described by Sui *et al*.^23^, which used probes with 18 nt initiator sequences, in combination with sets of 36 nt orthogonal hairpin amplifiers. We synthesized DNA oligonucleotide probes that contain a 20 nt sequence complementary to a miRNA of interest, flanked by two 18 nt initiators, connected by two base linkers (Fig. 1a, Supplementary Table S1). miRNA probes were hybridized to tissue sections prepared from fixed mouse retinas, then washed at high stringency using tetramethyl ammonium chloride (TMAC) for sequence specificity, as we have described previously^6,17^. Probes were detected by HCR using 36 nt hairpin amplifiers labeled with Cy3 (Supplementary Fig. S1, Supplementary Table S2). Probes for two different retinal miRNAs, miR-182 and miR-183, which are produced from the same primary transcript^28,29^, generated predominantly cytoplasmic fluorescent signals in cell bodies of photoreceptors in outer nuclear layer (ONL) and in cells located in the outer part of the inner nuclear layer (INL; Fig. 1d–f,n). miR-182 and miR-183 differ at only three positions within the probe complementary sequences (Fig. 1a). A probe that was mismatched with both miRNAs at those three positions generated little signal, similar to a control probe which contained only the two initiators joined by a 4 nt linker, or a no probe control (Fig. 1g–i). The expression patterns for miR-182 and miR-183 detected by miRNA *in situ* HCR are essentially identical to the expression patterns detected by an alkaline phosphatase (AP) conjugated antibody after *in situ* hybridization with a hapten-labeled RNA oligonucleotide probe (Fig. 1b,c), using our previous method^6^. To test simultaneous detection of two miRNAs by *in situ* HCR, we synthesized a miR-182 probe with a different pair of initiator sequences, compatible with a second set of hairpin amplifiers labeled with Cy5. The new miR-182 probe was hybridized simultaneously with the miR-183 probe, and the probes were detected simultaneously with the two sets of hairpin amplifiers. The resulting Cy3 and Cy5 fluorescent signals labeled same retinal cells (Fig. 1j–m,o), although many of the individual spots of Cy3 or Cy5 signal within the same cell were distinct and not overlapping (Fig. 1o), consistent with the detection of independent hybridization events for each miRNA probe. Detection of other miRNAs in the retina by *in situ* HCR yielded distinct expression patterns. Let-7f, a widely expressed miRNA, was detected in most retinal cells, but with less signal in the outer nuclear layer (Fig. 1p,q). In contrast, miR-126-3P, a miRNA specifically expressed in vascular endothelial cells^30,31^ strongly labeled retinal blood vessels, as well as cells in the choroid (Fig. 1r–u).

**Figure 2:**
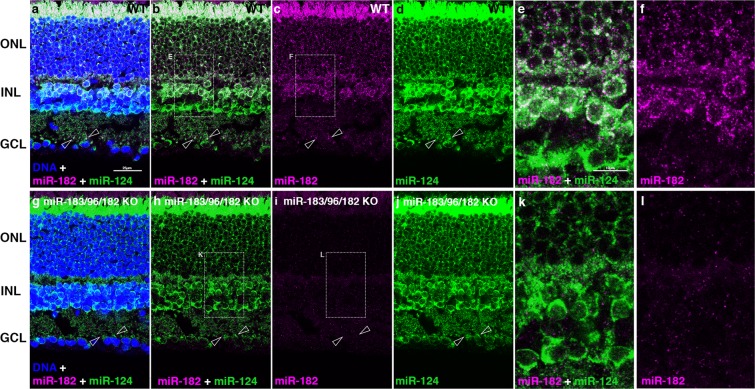
Detection of miR-182 by *in situ* HCR is abolished in miR-183/96/182 mutant mice. Retina sections from adult wild-type (**a**–**f**) or mice with disrupted miR-183/96/182 host gene (**g**–**l**) hybridized with probes for miR-182 and miR-124. (**e**,**j**,**k**,**l)** enlargements of regions indicated in b, c, h, i. (**a**–**d**) Arrowheads indicate overlapping localized miR-124 and miR-182 expression within the IPL; miR-124 expression is detected in the IPL for retinas with the disrupted miR-183/96/182 host gene, but colocalized miR-182 is absent (**g**–**j**). Magenta: Cy3, green: Cy 5, blue: DNA. Scale bars: 25 µm (**a-d**,**g**–**j**); 10 µm (**e**,**f**,**k**,**l**).

To further test miRNA *in situ* HCR specificity, we hybridized a probe for miR-182 to adult retina sections prepared from mice with a homozygous disruption of the gene encoding the miR-183/96/182 primary transcript^32^ or matched wild-type controls. The miR-182 fluorescent signal was present in wild-type control retina sections, but absent from mutant retina sections (Fig. 2). We simultaneously hybridized and detected a probe for miR-124, an abundant neuron-specific miRNA expressed in most mammalian neurons including retinal neurons^6,29^. The miR-124 signal was similar in sections from both wild-type and mutant retinas (Fig. 2), with widespread expression in all retinal layers. Cells with miR-124 signal overlapped with miR-182 in wild-type retinas, but miR-124 was also detected in additional cells in the inner nuclear layer (INL) and ganglion cell layer (GCL) that did not express miR-182. In addition to the miR-182 signal present in the cell bodies, overlapping miR-182 and miR-124 signals were detected in the inner plexiform layer (IPL), suggesting localization of miR-182 and miR-124 to bipolar axons and/or synapses. In the mutant retinas, the miR-182 signal is absent from the IPL (Fig. 2b,c,h,i).

### Combined miRNA and mRNA *in situ* HCR for identification of miRNA expressing cells

**Figure 3:**
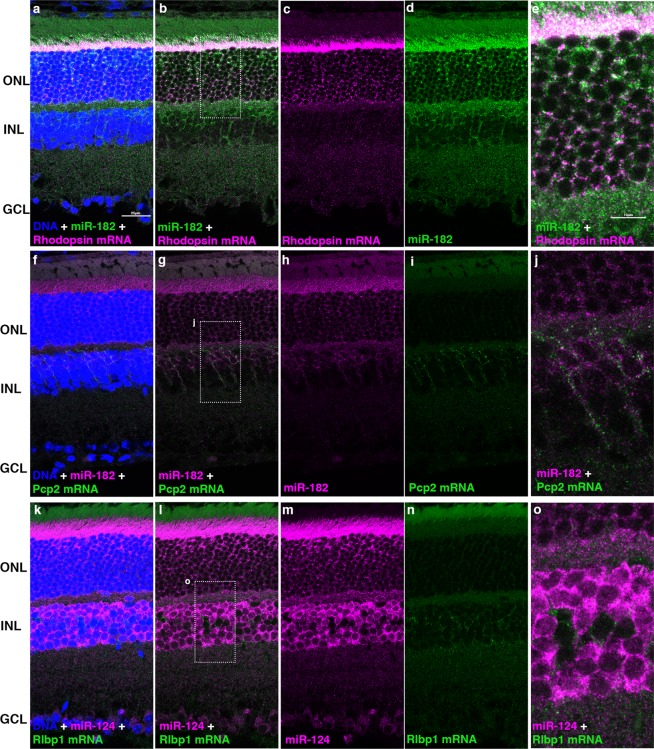
Combined detection of miRNAs and mRNAs by *in situ* HCR for cell identification. (**a–e**) miR-182 expression overlaps with Rhodopsin (Rho) expression in mouse rods. (**f**–**j**) miR-182 expression also overlaps with Pcp2, a marker of rod and ON cone bipolar cells. (**k**–**o**) miR-124 is absent in Müller glial cells expressing Rlbp1. (**e**,**j**,**o**) show enlargements of the indicated regions in b, g, l. Magenta: Cy3, green: Cy5, blue: DNA. Scale bars: 25 µm (**a**–**d**,**g**–**i**,**k–m**); 10 µm (**e**,**j**,**k**).

The ability to detect miRNAs and mRNAs in the same tissue section by *in situ* HCR would facilitate the identification and analysis of miRNA expressing cells. Sections from retinas were sequentially hybridized first with a pool of 2–5 HCR probes complementary to a specific mRNA (Supplementary Fig. S1, Supplementary Table S3) and then with a miR-182 or miR-124 probe with distinct initiators. Probes were simultaneously detected using Cy3 and Cy5 labeled hairpin amplifiers. We combined probes against Rhodopsin (Rho), an mRNA expressed exclusively in rod photoreceptors, with a probe to miR-182. As expected, both the miRNA and Rho mRNA could be detected in the rod photoreceptor cells (Fig. 3a–e) of the outer nuclear layer (ONL). miR-182 also was detected in cells of the INL that were negative for Rho mRNA. We hybridized the miR-182 probe in combination with a probe set against Pcp2, a mRNA expressed in rod bipolar cells and ON cone bipolar cells^33^ and observed overlapping expression in cells of the outer INL. The probe for Pcp2 labeled both bipolar cell bodies and presumptive axons projecting to the IPL (Fig. 3f–j). Strikingly, the miR-182 probe often appeared to label the same processes, although the individual spots of signal for each probe rarely overlapped (Fig. 3j). We also compared the expression of Rlbp1, an mRNA expressed in Müller glial cells^34^ with miR-124 (Fig. 3k–o). A small subset of cells in the central region of the INL appeared negative for miR-124, and those cells were positive for Rlbp1. These observations indicate that miR-124 is specifically expressed in retinal neurons, but not in Müller glia, consistent with prior observations^35,36^.

### Characterization of miR-181a expressing cells in the developing and adult mouse retina

**Figure 4:**
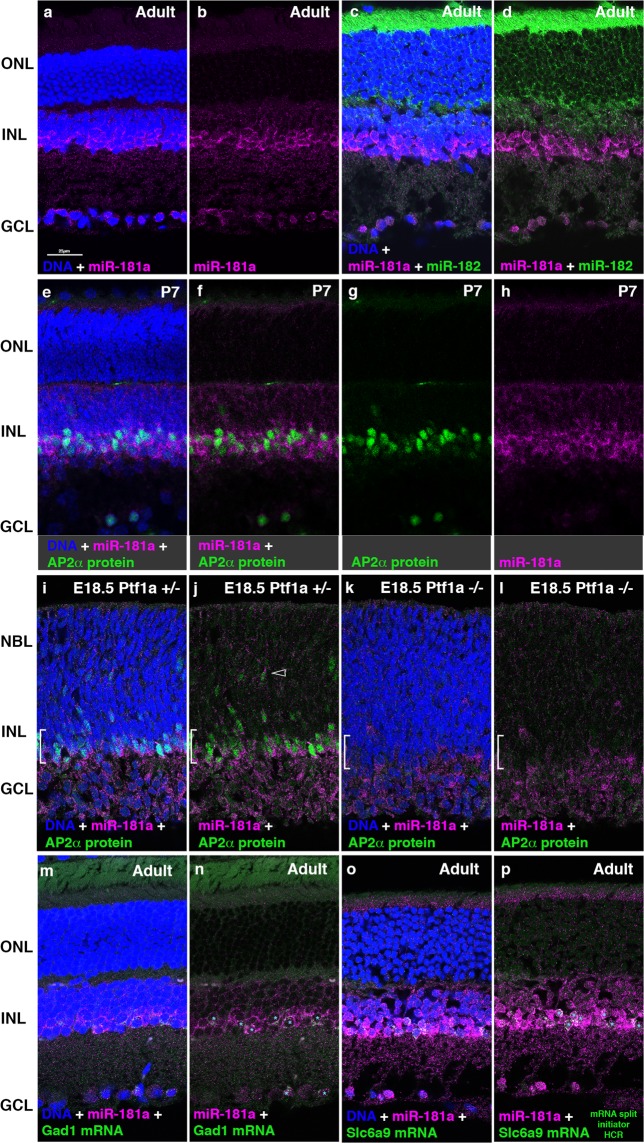
Characterization of miR-181a expressing retinal cells. (**a**,**b**) miR-181a is highly expressed in inner INL of in adult mouse retinas. (**c**,**d**) High level miR-181a and miR-182 expression does not overlap, although lower level signal for miR-181a overlaps with miR-182 in the INL. (**e**–**h**) miR-181a detected by *in situ* HCR and the amacrine cell specific protein AP2α detected by indirect immunofluorescence in P7 retina sections. Cells with strongest miR-181a signal overlap AP2α. (**i**–**l**) AP2α protein and strong expression of miR-181a overlap at E18.5 in forming amacrine layer, but both are lost in Ptf1a −/− retinas, although lower level signal for miR-181a persists in the GCL and INL. (**m**,**n**) Combined detection of miR-181a with Gad1 mRNA in GABAergic amacrine cells. (**o**,**p**) Combined detection of miR-181a and Slc6a9a mRNA in glycinergic amacrine cells, using split initiator HCR probes for Slc6a9 mRNA *in situ* HCR. Complete sets of panels for m-p are shown in Supplementary Figs. S2 and S3. Examples of overlapping cells in n and p indicated by cyan asterisks. Magenta; Cy3, green: Alexa-647 (**e**–**l**) or Cy5 (**m**,**n**) or Alexa-488 (**o**,**p**), blue: DNA. Scale bar: 25 µm (**a**–**p**).

miR-181a has been reported to be expressed in the INL and the GCL in the mouse retina^37^, but its expression pattern has not been characterized in detail. We detected miR-181 by *in situ* HCR in the INL and GCL of adult or P7 retinas, with the highest signal in presumptive amacrine cells located in the inner part of the INL, and no signal in the ONL (Fig. 4a,b,e–h). Combined detection of miR-181a and miR-182 in the adult retina showed distinct expression patterns, with overlap between low level miR-181a signal and miR-182 in the bipolar cells of the INL (Fig. 4c,d). AP2α (Tfap2a) is a transcription factor expressed in most amacrine cells^38^. After *in situ* HCR for miR-181a, we processed sections from P7 mouse retinas for indirect immunofluorescence using an antibody against the AP2α protein (Fig. 4e–g). Nuclear expression of AP2α protein overlapped cells with the strongest signals for miR-181a in both the INL and GCL, indicating that the retinal cells with the highest levels of miR-181a are amacrine cells or displaced amacrine cells. These observations demonstrate that it is possible to combine indirect immunofluorescence detection of proteins with miRNA detection by *in situ* HCR.

*In situ* HCR detection of miR-181a combined with indirect immunofluorescence detection of AP2α protein was also applied to embryonic retina sections from E18.5 mice heterozygous or homozygous for replacement of the Ptf1a transcription factor coding region with Cre^39^. Loss of both alleles of Ptf1a prevents the formation of nearly all amacrine and horizontal cells in the retina^40^, and also leads to death shortly after birth. Retinas from Ptf1a heterozygous embryos had a strong signal for miR-181a at the inner edge of the neuroblast layer and in a few scattered cells in the neuroblast layer, which overlapped with AP2α expression (Fig. 4i,j), likely newly generated amacrine cells that are migrating inward. These results are consistent with the embryonic generation of most amacrine neurons^41,42^. In contrast, both AP2α and the strong signal for miR-181a were absent in Ptf1a null retinas (Fig. 4k,l), consistent with the loss of amacrine cells. However, a widespread lower level signal for miR-181a was still present in the inner layers of the Ptf1a null retinas, consistent with the lower level signal for miR-181a in cells negative for AP2α in the INL and GCL of wild-type retinas at later ages (Fig. 4b,h).

We combined miR-181a *in situ* HCR with mRNA detection by *in situ* HCR to further characterize miR-181a expressing amacrine cells. Multiple subtypes of amacrine neurons have been identified, based on neurotransmitter use, molecular markers, and/or morphology^43^. In the rodent retina, the two most abundant subsets of amacrine cells use either GABA or glycine as a neurotransmitter. GABAergic amacrine cells can be identified by the expression of Gad1 mRNA^3,44^. We combined detection of miR-181a by *in situ* HCR with the detection of Gad1 mRNA by *in situ* HCR (Fig. 4m,n, Supplementary Fig. S2). Gad1 mRNA was present in the inner part of the INL, in a subset of the cells that were strongly labeled by miR-181a, indicating that miR-181a is expressed in GABAergic amacrine cells. The development of paired probes with split initiators is a recent improvement for mRNA *in situ* HCR^19^. Two 25 nt probes bound at adjacent positions on a target mRNA each contribute half of a 36 nt initiator sequence, so that a functional initiator is available for HCR only when both probes are bound in close proximity (Supplementary Fig. S1). The use of the two-component initiator reduces background and allows the use of larger probe pools against a target mRNA (10 or more probe pairs). Since both the probes and the half initiator sequences are similar in length to our miRNA *in situ* HCR probes, we tested whether this method could be combined with miRNA *in situ* hybridization under the same hybridization and wash conditions. We simultaneously hybridized and detected the probe for miR-181a in combination with 11 pairs of split initiator probes (Supplementary Table S4) for the Slc6a9 mRNA, which encodes Glycine Transporter-1, a marker of glycinergic amacrine cells^3,45^. Probe pools containing only one probe from each pair of split initiator probes were used as controls^19^. The mRNA probes were detected using 72 nt hairpin amplifiers at the same time as miR-181a was detected with 36 nt hairpin amplifiers. We observed clear overlap between the signals for miR-181a and Slc6a9 mRNA, indicating the miR-181a is expressed in glycinergic amacrine cells (Fig. 4o,p and Supplementary Fig. S3). Taken together, these results indicated that miR-181a is expressed in two major subpopulations of retinal amacrine cells.

## Discussion

Our results demonstrate that *in situ* HCR can be used effectively for the detection of endogenous miRNAs in fixed retinal tissue. At least two different miRNAs can be simultaneously detected by *in situ* HCR, using orthogonal hairpin amplifiers. Furthermore, we show that miRNA *in situ* HCR can be combined with *in situ* HCR detection of mRNAs, or with immunofluorescent antibody detection of proteins. The application of *in situ* HCR to miRNA detection should facilitate the identification and characterization of cells expressing specific miRNAs. Combined miRNA and protein detection have been used previously to identify miRNA expressing cells in the retina, using miRNA *in situ* hybridization with a fluorescent enzymatic detection method and antibody detection of the protein^15^ as well as non-neural tissues^14^. The ability to combine miRNA detection with mRNA detection by *in situ* HCR provides additional options for cell identification and characterization. The recent development of methods such as seqFISH^1^ or single cell RNA-seq.^3^ allow extensive analysis of mRNA expression in individual cells. However, seqFISH or single cell sequencing analysis of mature miRNA expression are not currently feasible. The ability to compare miRNA and mRNA expression by *in situ* HCR should help to fill this gap. Although HCR has not been deployed for *in situ* detection of miRNAs in tissue previously, HCR based methods have been used for miRNA detection^24,25,46–49^. While we have focused on the analysis of miRNAs in the retina, we expect that the miRNA *in situ* HCR method should be applicable for other tissues, as *in situ* HCR methods have been utilized for the detection of mRNAs in a wide variety of tissues and organisms^20^.

The retinal expression patterns for miR-182 and miR-183 detected by *in situ* HCR are consistent with prior analyses^28,32^, as well as with miRNA *in situ* hybridization based on our previous method using fluorescein-labeled RNA probes and an AP-conjugated anti-fluorescein antibody. Combined miRNA and mRNA *in situ* HCR allowed us to readily compare miR-182 and miR-124 expression with mRNA markers for cell types that either overlapped or were distinct from the miRNA expressing cells. Detection of miR-124 expression in retinal neurons, and miR-126-3p in blood vessels are consistent with prior reports^6,14,30,36^.

miR-181a regulates amacrine and ganglion cell process outgrowth in fish^50,51^, but it has not been well characterized in mammalian retinas. In the mouse retina we found that miR-181a was present in the INL and GCL of the mouse retina, but absent from ONL, starting during development and continuing in the adult. The strongest signal for miR-181a was in retinal amacrine cells, based on overlap with the transcription factor AP2α, although lower level signals were present in other cells of the INL and GCL. These results are consistent with a previous report of miR-181a expression in the INL and GCL of the P7 mouse retina by *in situ* hybridization^37^, and are similar to miR-181a expression in Medaka retina^50^. High level miR-181a signal was absent in embryonic retinas from Ptf1a null mice, which lack amacrine cells^40^, but a lower level miR-181a signal remained in the inner retina, consistent with lower level miR-181a expression in other cell types. The majority of amacrine cells use either GABA or glycine as a neurotransmitter, with most GABAergic amacrine cells generated before birth^52^. Since we observed widespread miR-181a expression in amacrine cells starting before birth, we compared miR-181a expression to mRNA markers specific for these two amacrine cell subpopulations in adult retinas. High level miR-181a signal overlapped with markers for both glycinergic and GABAergic amacrine cells, indicating that miR-181a is expressed in both of these major subsets of amacrine cells.

The ability to combine detection of mRNA markers with miRNA detection facilitates the analysis of miRNA expression in specific cell types as we have shown here for retina, and as previously reported in brain using a different method^16^. In addition, it should allow more precise expression comparisons between miRNAs and candidate target genes. In tissues with complex cell populations, it can be challenging to determine whether a miRNA and a potential target mRNA are expressed in the same or different subsets of cells. miRNA *in situ* HCR combined with mRNA *in situ* HCR can resolve overlapping or distinct expression. Analysis of miRNA expression by *in situ* hybridization studies often uses AP detection with a visible precipitate. While sensitive and easy to visualize, it can be difficult to combine visible AP staining with other detection methods for mRNA or proteins (e.g. the precipitate can block fluorescence excitation). miRNA *in situ* methods with fluorescent detection suitable for combined detection have been described, based on enzymatic amplification^10,11,14–16,53^, or a combination of proprietary nucleic acid amplification and enzymatic amplification (RNAscope, which is capable of combined detection of a miRNA with proteins or mRNAs^13,54^). However, inactivation of enzymes during sequential enzymatic amplification for double label RNA detection may require harsh conditions^55^, and commercial RNAscope reagents are relatively expensive. The ability to use miRNA *in situ* HCR for combinatorial miRNA detection or in combination with mRNA or protein detection should provide a flexible alternative.

The conditions we employ for miRNA *in situ* HCR yield are highly sequence specific^6,17^, yielding a low signal from probes with mismatches (Fig. 1). An alternate approach for sequence specific miRNA probes has been to incorporate LNA bases into probes^9^. However, different LNA probes often require different hybridization or wash temperatures to discriminate between related sequences^11,15,56^. The miRNA *in situ* HCR method employs the same hybridization and wash temperature for multiple probes, and these temperatures are lower than typically used with LNA probes. The milder hybridization/wash and gentle HCR detection conditions likely help to retain miRNAs in tissue sections without the need for additional fixation^11^, and may help to preserve proteins and mRNAs. However, a proteinase K digestion step is used to improve tissue access, which may damage or remove some protein antigens. Also, custom synthesized unmodified DNA oligonucleotide probes are widely available and less expensive than either RNA or LNA-modified probes, which should facilitate the use of the HCR method.

Recently an improved 3^rd^ generation HCR *in situ* method for mRNAs has been described^19^, in which binding of paired probes at adjacent locations on a target RNA creates a composite initiator sequence that can be bound by the HCR amplifier hairpins. The use of paired probes reduces background, improving the signal to noise for mRNA detection, so that larger pools of probes can be used to increase mRNA detection sensitivity. We found that mRNA detection by split initiator *in situ* HCR methods can be combined with miRNA *in situ* HCR in a single protocol with all probes hybridized, washed, and detected simultaneously. Unfortunately, it appears infeasible to apply the split initiator method for the detection of miRNAs, as miRNAs are not long enough to allow for the binding of two adjacent paired probes with sufficient length to maintain specificity. However, the relatively high abundance per cell of many miRNAs still allows miRNA detection with low background, using only a single HCR probe as we show here. For low level miRNAs, two rounds of HCR^57^ or an additional coupled enzymatic amplification (e.g. via an antibody conjugate against a hapten on the HCR amplifiers) could improve detection.

## Materials and Methods

### HCR *in situ* probes and hairpin amplifiers

DNA oligonucleotides were purchased from Integrated DNA Technologies (IDT); sequences are in Supplementary Tables S1–S4. Probes were gel purified prior to use. HCR DNA probes are 60 nt long including an 18 nt HCR initiator at 5′ end, 2 nt spacer, 20 nt miRNA recognition sequence complementarity to a specific target miRNA, 2 nt spacer, and a second 18 nt HCR initiator at 3′ end. The miR-124 probe contains only a 19 nt miRNA recognition sequence because of its higher GC content. HCR 36 nt hairpin amplifiers were HPLC purified. Hairpins H1 and H2 (Cy3) correspond to the “H1/H2 for OT” HCR amplifiers in^23^, while hairpins H3 and H4 (Cy5) correspond to the “H1/H2 for VP” HCR amplifiers in^23^. For split initiator mRNA *in situ* HCR^19^, a pool of 11 probe pairs was designed for Sl6a9. Matching 72 nt hairpin amplifiers (Set B3 with Alexa-488 label^21^) were purchased from Molecular Technologies.

### Animals and tissue preparation

Animal experiments were approved by the University of Michigan Institutional Animal Care & Use Committee and were carried out in accordance with relevant guidelines and regulations. Wild-type retinas were isolated from CD-1 mice (Charles River). Ptf1a mutant mice^39^ have the Ptf1a coding region replaced with the Cre coding region. Retinas were prepared by fixation of dissected mouse eyes for 30 minutes in 2% paraformaldehyde (PFA) in phosphate buffered saline (PBS) at room temperature, rinsed in PBS, dehydrated in 30% sucrose, and embedded in OCT. 14 µm sections were cut on a cryostat, transferred to Superfrost/plus microscope slides, and stored at −80 °C. For Fig. 4c,d, retinas were fixed for 75 minutes in 2% PFA in PBS. For Fig. 2, retinas from 8 week old miR-183/96/182 knockout (miR-183C^GT/GT^) or age-matched wild-type control mice on a C57BL/6 J and 129 S mixed background were prepared as described previously^32^.

### miRNA *in situ* hybridization with RNA probes

20 nt RNA probes (Sigma; Supplementary Table S5) include fluorescein modifications at both 5′ and 3′ ends and were gel purified prior to use. miRNA *in situ* hybridization with the fluorescein-labeled RNA probes and an AP-conjugated anti-fluorescein antibody (Roche) was performed essentially as described previously^6^.

### miRNA and mRNA *in situ* HCR

Detailed protocols for miRNA and mRNA *in situ* HCR are included in the Supplementary Methods. For miRNA *in situ* HCR, processing up to probe hybridization was essentially the same as described previously^6^. In brief, tissue sections were post-fixed with 4% paraformaldehyde, treated with Proteinase K, re-fixed with 4% paraformaldehyde, and acetylated. Slides were then pre-hybridized at 37 °C for 2.5 hrs in hybridization buffer containing 15% formamide, 5X SSC, 0.3 mg/ml yeast RNA, 100 µg/ml heparin, 1X Denhardt’s solution, 0.1% Tween 20, 0.1% CHAPS, 5 mM EDTA, and 300 nM 12mer random DNA primers. One or two miRNA probes (100 nM per probe) were hybridized overnight at 37 °C in hybridization buffer. For miRNA combined with mRNA *in situ* HCR using split initiator probes, a pool of 11 probe pairs replaced one of the miRNA probes, at a final concentration of 5 nM for each individual probe. After hybridization, slides were rinsed in 2X SSC with 0.1% Tween 20 (2X SSCT), then washed 3 times with 2X SSCT at 37 °C for 10 minutes each, followed by a high stringency wash in 3 M TMAC, 0.2% Tween 20, 50 mM Tris-HCl pH 8.0 at 45 °C for 30 minutes. Slides were then washed twice in 5X SSCT for 10 minutes at room temperature.

For miRNA combined with mRNA *in situ* HCR with two initiator probes, after prehybridization, 2–5 mRNA probes at 100nM-200nM total concentration (Pcp2, Gad1, Rho: 200 nM total, split equally among the individual probes: 40 nM per probe for a 5 probe pool; Rlbp1: 100 nM total) were hybridized overnight in hybridization buffer (same as above except with 50% formamide), then washed at 37 °C sequentially in 2X SSCT, 1X SSCT, 0.5X SSCT, and 0.25X SSCT for 15 min. each. Slides were next pre-hybridized at 37 °C in hybridization buffer (with 15% formamide) for 2.5 hrs, then hybridized overnight at 37 °C with one miRNA probe (100 nM) in hybridization buffer (with 15% formamide). Slides were washed as described above for miRNA probes.

For HCR detection, conditions were based on^20,23^. All steps were done at room temperature. Slides were washed in 5X SSCT for 10 min. Slides were incubated in amplification buffer (5X SSCT, 0.1% Tween 20, 10% low molecular weight dextran sulfate) at room temperature for 30 min., then incubated with hairpin amplifiers in amplification buffer (12pmol of each amplifier per 200 µl). Hairpin amplifiers were denatured at 95 °C for 90 seconds and cooled to room temperature prior to dilution in amplification buffer. Slides were rinsed briefly once in 5X SSCT, then washed 3 times in 5X SSCT for 15 min. each. For DNA staining, slides were incubated with Hoechst dye (2 µg/ml in 5X SSCT) for 45 min., then washed twice in 5X SSCT for 10 min. Slides were mounted in Aqua-Poly/Mount (Polysciences).

### Combined miRNA *in situ* HCR and immunofluorescence

We performed indirect immunofluorescence analysis after miRNA *in situ* HCR. After HCR detection, but prior to Hoechst staining, washed sections were incubated in blocking solution (1:400 unlabeled affinity purified Fab fragment donkey anti-mouse IgG (H + L) and 5% donkey serum in TBS: 100 mM Tris-HCl, pH 7.5, 150 mM NaCl, 0.05% Tween 20). Sections were then incubated with mouse anti-AP2α^38^ (Developmental Studies Hybridoma Bank) 1:1000 in TBS at room temperature for two hours. The sections were washed in TBS 3 times, then incubated with Alexa Fluor 647-conjugated anti-mouse secondary antibody (1:1000, Jackson Immunoresearch) for one hour at room temperature. Sections were washed in TBS 3 times and mounted in Aqua-Poly/mount.

### Microscopy and image processing

Sections were imaged on an Olympus FV1000 confocal microscope. Images were analyzed and colors were assigned with Fiji/ImageJ. Image brightness for miR-124 was reduced to balance channels in Fig. 2a,b,e,g,h,k. Representative images were cropped, assembled, and labeled using Adobe Photoshop.

## Supplementary information

Supplementary Methods and Figures.

Supplementary Tables.

## Data Availability

The raw data for this study is available upon reasonable request from the corresponding author.
