# Supplementary material for: Combined microRNA and mRNA detection in mammalian retinas by in situ hybridization chain reaction
Source: Sci Rep. 2020 Jan 15;10:351. doi: 10.1038/s41598-019-57194-0 (PMC6962165; doi:10.1038/s41598-019-57194-0)
Supplement: Supplementary file 1 — Supplementary Methods and Figures. [file 41598_2019_57194_MOESM1_ESM.pdf]

Combined microRNA and mRNA detection in mammalian retinas by in situ hybridization chain reaction

Pei Zhuang, Huanqing Zhang, Ryan M. Welchko, Robert C. Thompson, Shunbin Xu, and David L. Turner

Supplementary Materials and Methods

HCR Probe Design

For miRNAs, we generally used a 20 nt sequence complementary to the miRNA. The 2 nt junctions between the probe sequence and the initiators were adjusted to not be complementary with the target miRNA. Initiator sequences were oriented so that the polymer of hairpin amplifiers bound to each initiator would extend away from the bound probe sequence. For mRNA probes with two initiators, non-overlapping 45 nt target sequences in the mRNA were selected based on GC content (57% to 63%). For split initiator probes, non-overlapping pairs of 25 nt sequences, separated by 2 nt were selected after filtering based on GC content (35-65%). Regions of target mRNAs with a high degree of homology to other mRNAs (closely related genes) were excluded for probe design.

Oligonucleotide Probe Purification

DNA and RNA probes were gel purified prior to use. For the split-initiator mRNA probes, odd numbered probes or even numbered probes were combined into two separate pools and each pool was gel purified separately. Probe oligonucleotides were separated on 10% TBE-Urea gels (ThermoFisher Scientific); gels were stained with SYBR gold (ThermoFisher Scientific, S11494) and appropriate bands isolated. 400 µl elution buffer (10 mM Tris-Cl, pH 8.5) was added to crushed gel slices, followed by elution for 4 hours at room temperature with rotation. Elution buffer and gel material were transferred to 0.22 µm cellulose acetate filter tubes (Costar 8160) and the gel material separated by centrifugation. Flow-through solution was transferred into a new tube, the probe was precipitated with isopropanol, washed, and resuspended in RNase-free water. Purified probe concentrations were determined using a Nanodrop.

Tissue fixation

Dissected mouse eyes were usually fixed for 30 min. in 2% paraformaldehyde (PFA) in phosphate buffered saline (PBS) at room temperature. Fixation for 75 minutes yielded similar results. Fixation in 4% PFA in PBS may slightly reduce signal. Fixation in 2% PFA for 30 min. may be preferred for some protein antigens detected by indirect immunofluorescence. After fixation, tissue was rinsed in PBS, dehydrated in 30% sucrose, then embedded in OCT and sectioned on a cryostat. Sections were transferred to Superfrost Plus microscope slides and stored at -80°C.

Solutions

20X PBS

NaCl 160.0 g
KCl 4.0 g
Na_2HPO4 28.8 g
KH_2PO4 4.8 g

Dissolve in 800ml of water. Adjust the pH to 7.4 with HCl.
Adjust final volume to 1 Liter.
For RNase-free PBS, treat with diethyl pyrocarbonate (DEPC) 0.1% overnight at 37°, then autoclave.
DEPC should be added in a fume hood with appropriate protective gear.

20X SSC

3.0 M NaCl
0.3 M Sodium Citrate, pH 7.0

For RNase-free SSC, treat with DEPC (0.1%) overnight at 37°, then autoclave.
DEPC should be added in a fume hood with appropriate protective gear.

5X SSCT

5X SSC
0.1% Tween 20

2X SSCT

2X SSC
0.1% Tween 20

PK buffer

50 mM Tris-HCl, pH 7.5
5mM EDTA

Proteinase K

Dilute stock solution to 10 microgram/ml in PK Buffer; store in aliquots at –20° C.

1M Triethanolamine (pH 8.0)

Add 66.5 ml Triethanolamine and 20 ml concentrated HCl to 413.5 ml RNase-free water.

Acetic Anhydride (Sigma, Catalog No. A6064)

100X Denhardt's solution

2% bovine serum albumin (BSA; ICN 810661)
2% Polyvinylpyrrolidone (PVP-40, Sigma)
2% Ficoll 400 (Sigma)

Prepare a slurry in RNase-free water and dilute to final concentration. Store at –20° C.

Hybridization Solution (15% FA) (50 ml)

Final concentration	*stock/volume to make 50 ml*
15% formamide	7.5 ml of formamide
5X SSC	12.5 ml of 20 X SSC
0.3 mg/ml yeast RNA	0.3 ml of 50 mg/ml in RNase-free water
100 microgram/ml Heparin	100 µl of 50mg/ml in RNase-free water
1X Denhardt's solution	0.5ml of 100X Denhardt's solution
0.1% Tween 20	0.5ml of 10% Tween 20 in RNase-free water
0.1% CHAPS	0.5ml of 10% CHAPS in RNase-free water
5 mM EDTA	0.5ml of 0.5 M EDTA pH 8.0
3 µl/ml random primer	Stock: 0.1 nm/µl random 12mer DNA oligonucleotides

Adjust volume to 50 ml with RNase-free water. Store at –80° C.

50% FA Hybridization Solution (50 ml)

Final concentration	*stock/volume to make 50 ml*
50% formamide	25 ml of formamide
5X SSC	12.5 ml of 20 X SSC
0.3 mg/ml yeast RNA	0.3 ml of 50 mg/ml in RNase-free water
100 microgram/ml Heparin	100 µl of 50mg/ml in RNase-free water
1X Denhardt's solution	0.5ml of 100X Stock
0.1% Tween 20	0.5ml of 10% Tween 20 in RNase-free water
0.1% CHAPS	0.5ml of 10% CHAPS in RNase-free water
5 mM EDTA	0.5ml 0.5 M EDTA pH 8.0
3 µl/ml random primer	Stock: 0.1 nm/µl random 12mer DNA oligonucleotides

Adjust volume to 50 ml with RNase-free water. Store at –80° C.

Note: The random sequence 12mer DNA oligonucleotides included in both hybridization solutions are for blocking potential nonspecific binding of probe oligonucleotides.

TMAC wash solution

3M Tetramethyl Ammonium Chloride (TMAC) (Sigma)
0.2% Tween 20
50 mM Tris-HCl, pH 8.0

TBS

100 mM Tris-HCl, pH 7.5
150 mM NaCl
0.05% Tween 20

HCR hairpin storage buffer

- 10 mM Tris-HCl, pH 8.0
- 300 mM NaCl
- 1 mM EDTA pH 8.0

HCR Amplification buffer

- 5X SSC
- 0.1% Tween 20
- 10% low MW dextran sulfate (Sigma D4911)

Slides, hybridization chambers, and mounting medium

Microscope slides

- Superfrost Plus (Fisher Scientific, Cat. #12-550-15)

Hybridization chamber coverslips

- CoverWell Incubation Chambers (Electron Microscopy Sciences, Cat. #70324-20)

Aqueous Mounting Medium

- Aqua-Poly/Mount (Polysciences, Inc.)

Protocols

1. microRNA in situ HCR for tissue sections with optional combined antibody staining

Methods for in situ HCR in Fig. 1, Fig. 2, Fig. 4a-l.

All steps are performed at room temperature (~20° C) unless indicated otherwise.

1. Warm slides at 37°C for 30 min.

2. Post-fix slides in 4% paraformaldehyde in PBS (make fresh) for 20 min.
   Perform this step in a fume hood with appropriate protective gear.

3. Wash slides twice in PBS for 10 min. each.

4. Treat the slides with 10 microgram/ml Proteinase K in PK buffer for 6 min. *(see note 1)*

5. Wash slides in PBS for 10 min.

6. Fix slides in 4% paraformaldehyde in PBS for 15 min.
   Perform this step in a fume hood with appropriate protective gear.

7. Wash slides in PBS for 10 min.

8. Rinse slides briefly in RNase-free water.

9. Acetylation: Incubate slides in 0.1M TEA + *acetic anhydride* for 5 min. twice.
   Immediately before transferring slides to the dish, add 0.5 ml acetic anhydride to 200ml 0.1M TEA (20ml 1M Triethanolamine (TEA) pH 8.0 + 180 ml RNase-free water). Mix with a pipet tip and/or by gentle rocking to disperse acetic anhydride. Transfer the slides as soon as acetic anhydride is dispersed.
   Perform this step in a fume hood with appropriate protective gear.

10. Wash slides in PBS for 10 min.

11. Pre-hybridize the slides in hybridization solution without probe.
   ~2.5 hr at 37°C in incubator (*precise time for pre-hybridization is not critical).*

12. Hybridize probe(s) on slides, using hybridization chamber coverslips:
   Add 20 pmol of each miRNA probe to 200 μl of hybridization solution. *(1 or 2 miRNA probes at final concentration of 100nM for each. See note 2)*

Pre-warm hybridization solution with probe(s) at 37°C for 10 min. before adding to slides.

Hybridize probe(s) overnight at 37°C for ~18 hours.
The temperature is important: higher temperatures may decrease signal.

13. Remove probe solution and rinse in 2X SSCT at 37°C

14. Wash three times in 2X SSCT at 37°C for 10 min. each.

16. High-stringency wash: incubate in TMAC wash solution at 45° C for 30 min.
Pre-warm the TMAC wash solution to 45° C prior to adding to slides. The precise temperature and duration of these washes is critical. We typically do this wash in a dish in a shaking water bath. Measure bath temperature to confirm the indicated temperature.
Note: TMAC is toxic. Handle with appropriate protective gear and perform this step in a fume hood.

18. Wash twice in 5X SSCT for 10 min. each.

HCR detection

19. Pre-amplify the slides in amplification buffer for 30 min.

20. Amplification (at room temperature):
Hairpin stock solution: 100 μM in hairpin storage buffer
Hairpin working solution: 6 μM of each hairpin amplifier in hairpin storage buffer

Prepare each hairpin pair by snap cooling 2 μl per slide of 6 μM working solution: heat at 95° C for 90 seconds and cool to RT in dark. Add amplification buffer to 200 μl final volume per slide.

Final concentration of each hairpin amplifier is 12 pmol/200 μl.

Incubate slides with fluorescently labeled hairpin amplifiers for 2 hours.

21. Rinse off excess hairpins using 5X SSCT

22. Wash slides three times in 5X SSCT for 10 min. each.

For combined antibody staining, skip steps 23-24 and proceed to step 25.

23. Stain nuclei for DNA (optional):
Dilute Hoechst dye to 2 microgram/ml in 5X SSCT. Add 200 μl of diluted dye to each slide and incubate for 45 min., then wash slides twice in 5X SSCT for 10 min. each.

24. Mount slides in Aqua-Poly/Mount.

Optional Antibody Staining:
(continued from step 22)

25. Blocking for mouse primary antibodies: Incubate the slides with blocking solution (5% donkey serum and 1:400 diluted unlabeled affinity purified Fab fragment donkey anti-mouse IgG (H+L) in TBS) for 1 hour.

26. Wash slides three times in TBS for 10 min. each.

27. Incubate with mouse primary antibody (anti-AP2α: diluted 1:1000 in TBS) for 2 hours, using hybridization chamber coverslips.

28. Wash slides three times in TBS for 10 min. each.

30. Incubate with secondary antibody (Alexa-647 conjugated donkey anti-mouse IgG: diluted 1:1000 in TBS) for 1 hour.

31. Stain nuclei for DNA (optional): Incubate with Hoechst dye (2 microgram/ml in TBS) for 45 min.

32. Wash slides three times in TBS for 10 min. each.

33. Mount slides in Aqua-Poly/Mount.

<u>2. microRNA in situ HCR for tissue sections</u>
<u>with combined split initiator mRNA in situ HCR</u>

Methods for in situ HCR in Fig. 4o, p and Supplementary Fig. S3

All steps are performed at room temperature (~20° C) unless indicated otherwise.

1. Warm slides at 37°C for 30 min.

2. Post-fix slides in 4% paraformaldehyde in <u>PBS</u> (make fresh) for 20 min.
   Perform this step in a fume hood with appropriate protective gear.

3. Wash slides twice in PBS for 10 min. each.

4. Treat the slides with 10 microgram/ml Proteinase K in <u>PK buffer</u> for 6 min. *(see note 1)*

5. Wash slides in PBS for 10 min.

6. Fix slides in 4% paraformaldehyde in <u>PBS</u> for 15 min.
   Perform this step in a fume hood with appropriate protective gear.

7. Wash slides in <u>PBS</u> for 10 min.

8. Rinse slides briefly in RNase-free water.

9. Acetylation: Incubate slides in 0.1M TEA + *acetic anhydride* for 5 min. twice.
   Immediately before transferring slides to the dish, add 0.5 ml acetic anhydride to 200ml 0.1M TEA (20ml 1M Triethanolamine (TEA) pH 8.0 + 180 ml RNase-free water). Mix with a pipet tip and/or by gentle rocking to disperse acetic anhydride. Transfer the slides as soon as acetic anhydride is dispersed.
   Perform this step in a fume hood with appropriate protective gear.

10. Wash slides in <u>PBS</u> for 10 min.

11. Pre-hybridize the slides in <u>hybridization solution</u> without probe.
    ~2.5 hr at 37°C in incubator (*precise time for pre-hybridization is not critical).*

12. Hybridize probe(s) on slides, using hybridization chamber coverslips:
    Add 20 pmol of miRNA probe and the pool of split initiator probes (1 pmol of each individual split initiator probe) to 200 μl of <u>hybridization solution</u>.
    (the microRNA probe is at a final concentration of 100nM. The split initiator mRNA probes are at a final concentration of 5 nM for each individual probe.)

Pre-warm hybridization solution with probe(s) at 37°C for 10 min. before adding to slides.

Hybridize probes overnight at 37°C for ~18 hours.
The temperature is important: higher temperatures may decrease signal.

13. Remove probes and rinse in 2X SSCT at 37°C

14. Wash three times in 2X SSCT at 37°C for 10 min. each.

16. High-stringency wash: incubate in TMAC wash solution at 45° C for 30 min.
Pre-warm the TMAC wash solution to 45° C prior to adding to slides. The precise temperature and duration of these washes is critical. We typically do this wash in a dish in a shaking water bath. Measure bath temperature to confirm the indicated temperature.
Note: TMAC is toxic. Handle with appropriate protective gear and perform this step in a fume hood.

18. Wash twice in 5X SSCT for 10 min. each.

HCR detection

19. Pre-amplify the slides in amplification buffer for 30 min.

20. Amplification (at room temperature):
Hairpin stock solution: 100 μM in hairpin storage buffer
Hairpin working solution: 6 μM of each hairpin amplifier in hairpin storage buffer

Prepare each hairpin pair by snap cooling 2 μl per slide of 6 μM working solution: heat at 95° C for 90 seconds and cool to RT in dark. Add amplification buffer to 200 μl final volume per slide.
Final concentration of each hairpin amplifier is 12 pmol/200 μl.

Incubate slides with fluorescently labeled hairpin amplifiers overnight.

21. Rinse off excess hairpins using 5X SSCT

22. Wash slides three times in 5X SSCT for 10 min. each.

23. Stain nuclei for DNA (optional):
Dilute Hoechst dye to 2 microgram/ml in 5X SSCT. Add 200 μl of diluted dye to each slide and incubate for 45 min., then wash slides twice in 5X SSCT for 10 min. each.

24. Mount slides in Aqua-Poly/Mount.

3. microRNA in situ HCR for tissue sections
combined with two-initiator mRNA probes for in situ HCR

Methods for in situ HCR in Fig. 3, Fig. 4m-n, Supplementary Fig. S2.

All steps are performed at room temperature (~20° C) unless indicated otherwise.

1. Warm slides at 37°C for 30 min.

2. Post-fix slides in 4% paraformaldehyde in PBS (make fresh) for 20 min.
   Perform this step in a fume hood with appropriate protective gear.

3. Wash slides twice in PBS for 10 min. each.

4. Treat the slides with 10 microgram/ml Proteinase K in PK buffer for 6 min. *(see note 1)*

5. Wash slides in PBS for 10 min.

6. Fix slides in 4% paraformaldehyde in PBS for 15 min.
   Perform this step in a fume hood with appropriate protective gear.

7. Wash slides in PBS for 10 min.

8. Rinse slides briefly in RNase-free water.

9. Acetylation: Incubate slides in 0.1M TEA + *acetic anhydride* for 5 min. twice.
   Immediately before transferring slides to the dish, add 0.5 ml acetic anhydride to 200ml 0.1M TEA (20ml 1M Triethanolamine (TEA) pH 8.0 + 180 ml RNase-free water). Mix with a pipet tip and/or by gentle rocking to disperse acetic anhydride. Transfer the slides as soon as acetic anhydride is dispersed.
   Perform this step in a fume hood with appropriate protective gear.

10. Wash slides in PBS for 10 min.

11. Pre-hybridize the slides in 50% FA hybridization solution without probe.
    ~2.5 hr at 37°C in incubator (*precise time for pre-hybridization is not critical).*

12. mRNA probe hybridization:
    Hybridize probe(s) on slides, using hybridization chamber coverslips:

Add 40 pmol of each mRNA probe pool in 200 μl total volume of 50% FA Hybridization Solution
(A pool of 2-5 probes at 200 nM final concentration for each mRNA probe pool; for a pool of 5 probes, each probe is at a final concentration of 40nM)

Pre-warm hybridization solution + probes @37°C for 10 min.

Hybridize mRNA probes overnight at 37°C for ~18 hours.

13. Remove mRNA probes and rinse in 2X SSCT at 37°C

14. Wash slides in 2X SSCT for 10 min. at 37°C
1X SSCT for 10 min. at 37°C
0.5X SSCT for 10 min. at 37°C
0.25X SSCT for 10 min. at 37°C
0.125X SSCT for 10 min. at 37°C

15. Pre-hybridize the slides in hybridization solution without probe.
~2.5 hr at 37°C in incubator (*precise time for pre-hybridization is not critical).*

16. miRNA probe hybridization using hybridization chamber coverslips:
Add 20 pmol of the miRNA probe to 200 μl of hybridization solution
(*the microRNA probe is at a final concentration of 100nM*).

Pre-warm hybridization solution with probe at 37°C for 10 min. before adding to slides.

Hybridize probe overnight at 37°C for ~18 hours.
The temperature is important: higher temperatures may decrease signal.

17. Remove probe and rinse in 2X SSCT at 37°C

18. Wash three times in 2X SSCT at 37°C for 10 min. each.

19. High-stringency wash: incubate in TMAC wash solution at 45° C for 30 min.
Pre-warm the TMAC wash solution to 45° C prior to adding to slides. The precise temperature and duration of these washes is critical. We typically do this wash in a dish in a shaking water bath. Measure bath temperature to confirm the indicated temperature.
Note: TMAC is toxic. Handle with appropriate protective gear and perform this step in a fume hood.

20. Wash twice in 5X SSCT for 10 min. each.

HCR detection

21. Pre-amplify the slides in amplification buffer for 30 min.

22. Amplification (at room temperature):

    Hairpin stock solution: 100 μM in hairpin storage buffer
    Hairpin working solution: 6 μM of each hairpin amplifier in hairpin storage buffer

    Prepare each hairpin pair by snap cooling 2 μl per slide of the 6 μM working solution: heat at 95° C for 90 seconds and cool to RT in dark. Add amplification buffer to final volume of 200 μl per slide.

    Incubate slides with fluorescently labeled hairpin amplifiers for 2 hours.
    Final concentration of each hairpin amplifier is 12 pmol/200 μl.

23. Rinse off excess hairpins using 5X SSCT

24. Wash slides three times in 5X SSCT for 10 min. each.

25. Stain nuclei for DNA (optional):

    Dilute Hoechst dye to 2 microgram/ml in 5X SSCT. Add 200 μl of diluted dye to each slide and incubate for 45 min., then wash slides twice in 5X SSCT for 10 min. each.

26. Mount slides in Aqua-Poly/Mount.

Protocol Notes

(1) Different batches of Proteinase K need to be titrated for best results. It may be easier to adjust the time of incubation while using a fixed concentration of Proteinase K. Too high Proteinase K concentration or incubation for too long with Proteinase K may cause sections to tear, lift off from the slides, or disintegrate during in situ HCR processing. Insufficient Proteinase K treatment may reduce signal, presumably because of reduced access for probes or detection reagents. Different tissues or fixation may require different Proteinase K treatments.

(2) It is possible to use 40 pmol of miRNA probe per slide (200nM final concentration = 2X standard concentration), which may modestly increase signal, but also may increase background.

Supplementary Figures

Supplementary Fig. S1. (a) Schematic diagram of miRNA in situ HCR. A miRNA probe is hybridized to a target miRNA. The probe includes two initiator sequences, one at each end. After hybridization and washing to remove unbound probes, the bound probe is detected with a pair of 36 nt HCR amplifiers, both labeled with the same fluor (either Cy3 or Cy5). Each hairpin amplifier that is base paired with a probe initiator sequence in the initial round exposes a free initiator sequence for the other hairpin of the pair. Additional hairpin amplifiers bind to the newly exposed initiators, and the process repeats to generate large polymers of hairpin amplifiers at each probe binding site. If two miRNA probes are used, two distinct sets of hairpin amplifiers, one labeled with Cy3 and the other with Cy5, can be used simultaneously to detect the two miRNA probes independently. (b) Schematic of mRNA in situ HCR using probes with an initiator sequence at each end. Probes are longer than the miRNA probes, requiring different hybridization and washing conditions, but the HCR detection process is the same as in (a). Only one mRNA probe is shown, but pools of up to 5 probes against the same mRNA can be hybridized and detected together to increase signal. For combined mRNA and miRNA in situ HCR, the longer mRNA probe pool is hybridized and washed first, then the miRNA probe is hybridized and washed. Both sets of probes are detected simultaneously. (c) Schematic of mRNA in situ HCR using split initiator probes. Two probes bind to a target mRNA at adjacent sites (separated by 2 nt) and each probe contributes half of an initiator sequence. After hybridization and washing, one hairpin amplifier binds to the composite initiator sequence from a bound probe pair. The initial hairpin amplifier binding exposes a new initiator which allows

HCR amplification to proceed. For the HCR step, the schematic shows two mRNA probe pairs, but pools of 10 or more probe pairs against the same target mRNA can be hybridized and detected together for increased sensitivity. For control hybridizations, a subset pool comprised of all the odd or all the even initiator probes from each pair is used. The split initiator probes are detected using a pair of 72 nt hairpin amplifiers labeled with Alexa 488. For combined mRNA and miRNA in situ HCR, a split initiator mRNA probe pool and a miRNA probe are hybridized, washed, and detected simultaneously, using a combination of 36 nt and 72 nt hairpin amplifier pairs. Note: the polymers in this figure are comprised of only small numbers of hairpin molecules, but the actual polymers generated by HCR can incorporate hundreds of hairpin amplifiers [17].

Supplemental Fig. S1

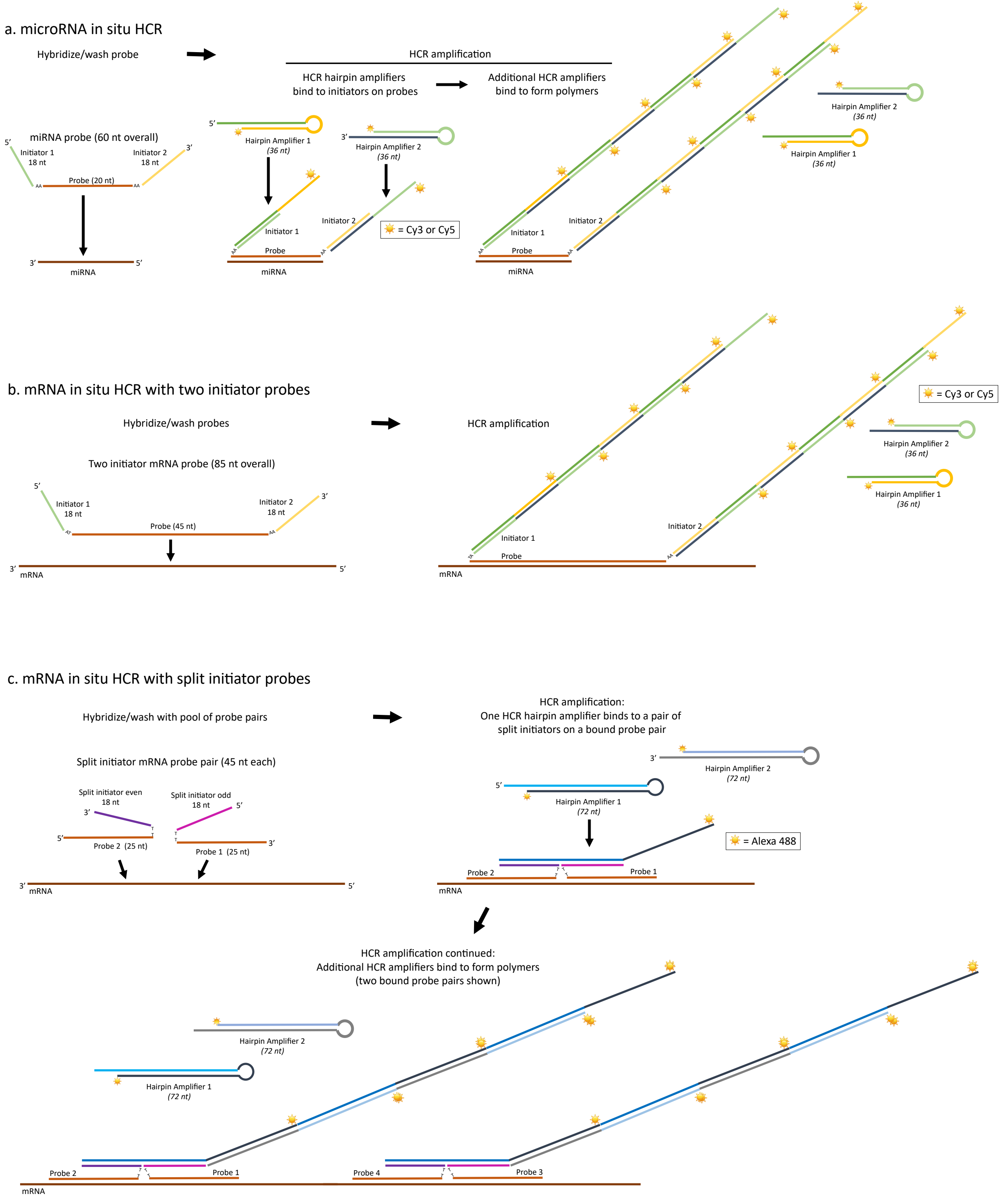

Supplementary Fig. S2. Complete set of panels for Fig. 4m, n showing combined detection of miR-181a with an mRNA specific for GABAergic amacrine cells (Gad1). Panels duplicated from Fig. 4 are indicated. Examples of overlapping cells indicated by cyan asterisks. Magenta: Cy3, green: Cy5, blue: DNA.

Fig. S2

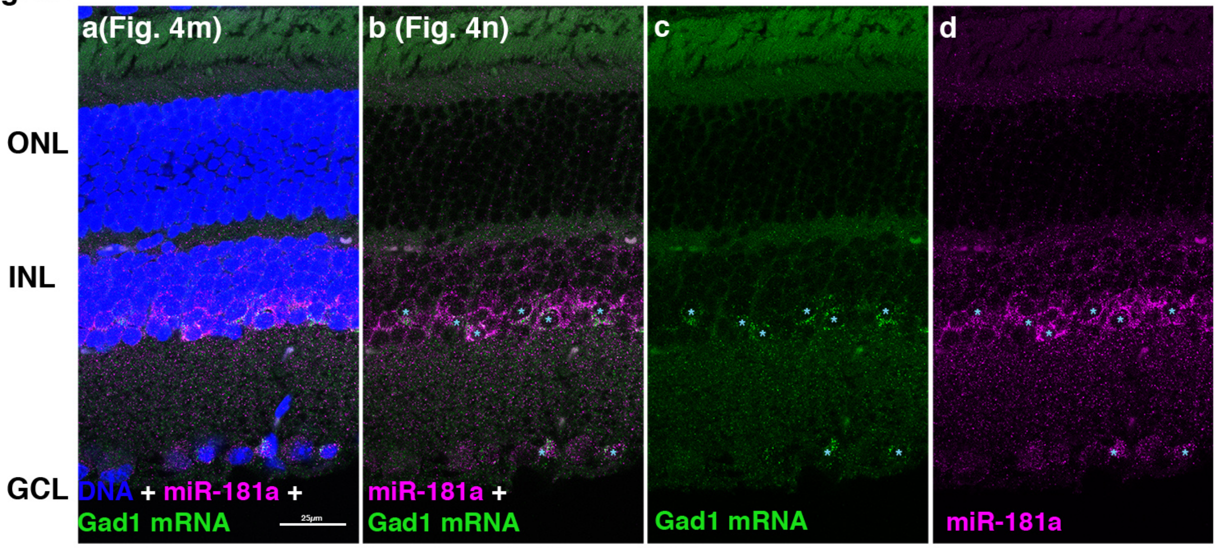

Supplementary Fig. S3. Detection of Slc6a9 mRNA by in situ HCR using split initiator probes, in combination with miR-181a. Includes the complete set of panels for Fig. 4o, p, as well as additional controls. (a-d) combined detection of miR-181a and Slc6a9 mRNA (a marker for glycinergic amacrine cells). Panels duplicated from Fig. 4 are indicated. Examples of overlapping cells indicated by cyan asterisks. (e-h) Control in situ HCR using the odd numbered subset of the Slc6a9 mRNA split probe pool, in combination with the miR-181a probe for miRNA in situ HCR. (i-l) Control in situ HCR using the even numbered subset of the Slc6a9 mRNA split probe pool, in combination with the initiator I1I2 only control probe for miRNA in situ HCR. Magenta: Cy3, green: Alexa-488, blue: DNA.

Fig.S3

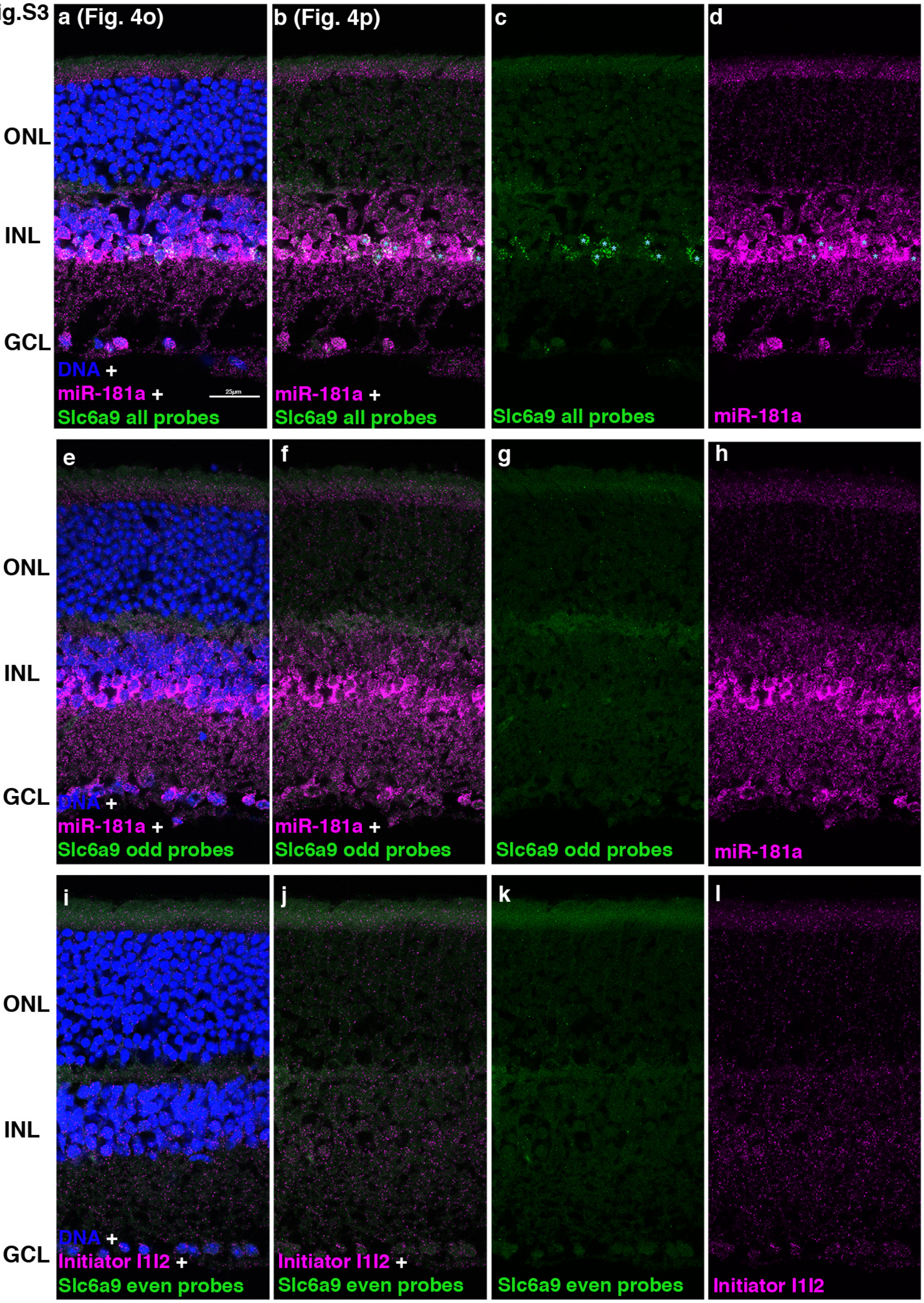
a (Fig. 4o)
b (Fig. 4p)
c
d
e
f
g
h
i
j
k
l
ONL
INL
GCL
DNA +
miR-181a +
Slc6a9 all probes
25µm
Slc6a9 odd probes
Slc6a9 even probes
miR-181a
Initiator I1I2 +
Initiator I1I2
